# Histologic Subtypes in Endometriosis-Associated Ovarian Cancer and Ovarian Cancer Arising in Endometriosis: A Systematic Review and Meta-Analysis

**DOI:** 10.1007/s43032-024-01489-9

**Published:** 2024-03-04

**Authors:** Francesca Chiaffarino, Sonia Cipriani, Elena Ricci, Giovanna Esposito, Fabio Parazzini, Paolo Vercellini

**Affiliations:** 1 Gynaecology Unit, Foundation IRCCS Ca’ Granda Ospedale Maggiore Policlinico, Via Commenda 12, 20122 Milan, Italy; 2 Department of Clinical Sciences and Community Health, University of Milan, Milan, Italy

**Keywords:** Endometriosis, Ovarian neoplasm, Histology

## Abstract

The definition of the association between ovarian cancer and endometriosis was first reported by Sampson in 1925. He identified the following criteria: (a) clear evidence of endometriosis in proximity to the tumour, (b) exclusion of a metastatic tumour to the ovary, (c) presence of tissue resembling endometrial stroma surrounding epithelial glands. The naming of these cancers is “endometriosis-associated ovarian cancer” (EAOC). Scott proposed an additional stringent criterion: evidence of histological transition from endometriosis to cancer is to define “ovarian cancer arising in endometriosis” (OCAE). The aim of this systematic review is to analyse the distribution of different ovarian cancer histotypes in EAOC and OCAE to understand their similarities and differences. A total of 31 studies were included. Four studies added data for both EAOC and OCAE. Twenty-three studies were selected for EAOC, with a total of 800 patients, and 12 studies were selected for OCAE, with a total of 375 patients. The results show no significant differences in the distribution of histotypes in the two populations analysed. Clear cell carcinoma (CCC) and endometrioid carcinoma (EC) were the most common subtypes and were less frequent in EAOC compared to OCAE; the odd ratios were 0.58 (0.26–1.29) and 0.65 (0.33–1.26) respectively, although the difference was not statistically significant. The other histotypes were present in small proportions. This analysis shows that the histological profiles of EAOC and OCAE are similar, suggesting a similar aetiopathological mechanism, which requires further research to investigate whether EAOC and OCAE may be in the same way but at different points of the process to malignancy or have different pathways of progression to malignancy.

**Supplementary Information:**

The online version contains supplementary material available at 10.1007/s43032-024-01489-9.

## Introduction

Endometriosis is a common inflammatory gynaecological disorder characterised by the presence of endometrial tissue outside the uterine cavity. Although endometriosis is considered a benign disease, malignant transformation has been reported. The most common site of malignant development is the ovary. Many studies have linked endometriosis to an increased risk of ovarian cancer; particularly, endometriosis is associated with a greater risk of clear cell and endometrioid histotypes, equal to 3.4-fold and 2.3-fold, respectively [1, 2]. However, the development and neoplastic transformation from endometriosis is usually controversial, and the epidemiological evidence on the association is still not clear.

Atypical endometriosis, characterised by endometriotic glands with cytological atypia, has been suggested as a transition between endometriosis and ovarian cancer associated with endometriosis [3]. In particular, atypical endometriosis, which occurs in 60–80% of ovarian cancers that result from endometriosis, is considered the true precursor of ovarian cancer [3, 4].

The definition of the association between ovarian cancer and endometriosis was first reported by Sampson in 1925 [5]. He identified the following criteria: (a) there must be clear evidence of endometriosis in proximity to the tumour, (b) exclusion of a metastatic tumour in the ovary, (c) presence of tissue resembling endometrial stroma surrounding epithelial glands. These cancers are called “endometriosis-associated ovarian cancer” (EAOC). Subsequently, Scott proposed an additional stringent criterion: evidence of histological transition from endometriosis to cancer to define “ovarian cancer arising in endometriosis” (OCAE) [6].

**Fig. 1:**
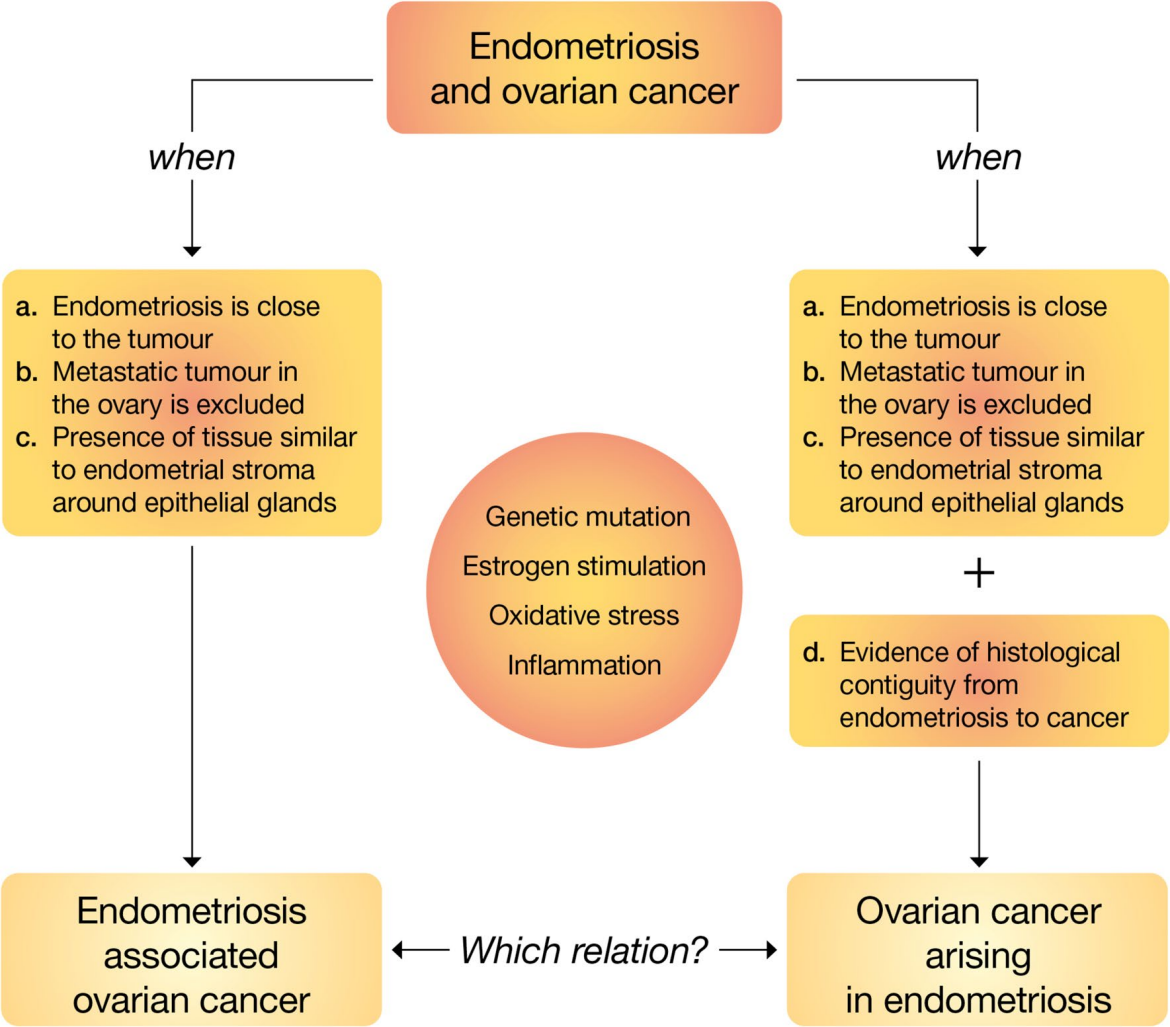
The endometriosis-associated ovarian cancer and the ovarian cancer arising in endometriosis

Several studies analysed data about EAOC, whereas few published studies fulfilled the application of the more stringent Scott’s criteria to define OCAE, and moreover, most articles regarding the malignant transformation of endometriosis are case reports. The real pathological and aetiological differences between these two populations remain unclear (Fig. 1).

It has been suggested, although not completely consistent, that EAOC patients tend to have a better prognosis than patients with ovarian cancer without endometriosis: differences in histology, tumour grade and early stage may explain these findings [7]. The prognosis and the histological characteristics of OCAE have rarely been studied [8].

To our knowledge, there are no studies that have evaluated the relation between EAOC and OCAE.

The aim of this systematic review is to analyse the distribution of different ovarian cancer histotypes in EAOC and in OCAE. Observing differences in the distribution of histological types of ovarian cancer in these two different anatomopathological conditions may provide some information to understand their similarities and differences.

## Methods

This review was conducted according to PRISMA (Preferred Reporting Items for Systematic reviews and Meta-Analyses) [9] and was registered in PROSPERO in April 2022 (Registration ID Number CRD42022314513).

### Literature Search

We performed a PubMed/EMBASE search of papers published from January 1990 to March 2022. The search terms “endometriosis”, “endometriotic cyst” and “endometrioma”, in combination with “ovarian neoplasm”, “ovarian cancer”, “endometrioid carcinoma” and “clear cell carcinoma”, were used as free text and as Medical Subject Heading (MeSH) terms.

A PICOS (Patient, Intervention, Comparator, Outcome, Study) design structure was used to develop the study questions and the inclusion/exclusion criteria.

### Study Selection

Two authors (F.C. and S.C.) screened the papers and independently selected the articles eligible for the systematic review and disagreements were resolved by discussion. They also reviewed the reference lists of the retrieved papers to identify any potential additional studies that could be included. If several published reports of the same study were available, only the one with the most detailed information was included. Only studies reporting original data, full-length articles and published in English were selected.

### Inclusion Criteria

The inclusion criteria for the selection of studies were as follows:

1. Studies in which the selection of cases of ovarian cancer with endometriosis met Sampson’s criteria and the cases were termed “endometriosis-associated ovarian cancer”.
2. Studies in which the selection of ovarian cancer cases with endometriosis also met the stricter Scott criteria and the cases were termed “ovarian cancer arising in endometriosis”.
3. Studies reporting the histotypes of ovarian cancer.
4. Studies reported the total number of ovarian cancers with endometriosis (without histotype selection) and number of different histotypes of ovarian cancer.

### Exclusion Criteria

Studies were excluded if they were:

1. Studies that included only some histological types or some stages of eligible patients
2. Studies that did not report the frequency of histological types

### Data Extraction Categories

For each study, the following information was collected: first author’s last name; year of publication; country of origin; study design; age, if available; endometriosis diagnosis and site; ovarian cancer histotype diagnosis; and FIGO (International Federation of Gynecology and Obstetrics) stage.

Supplementary file (SF) SF1 shows the flowchart of the studies selection.

### Statistical Analysis

Difference in EAOC and OCAE histological type distributions was evaluated with Pearson’s chi-square test.

Among the selected studies, four reported the histological type distribution of both EAOC and OCAE. With the aim to compare the frequency of endometrioid and/or clear cell histology in EAOC and OCAE, we computed the unadjusted odds ratios (OR) for each study. Then, random effect meta-analyses were performed to estimate overall unadjusted OR and corresponding 95% confidence interval. Percentages of residual variation due to heterogeneity were also reported.

Analyses were performed using STATA (StataCorp. 2015. Stata Statistical Software: Release 14. College Station, TX: StataCorp LP) and SAS 9.4 (SAS Institute, Cary, NC, USA).

## Results

A total of 31 studies were identified for inclusion in the systematic review. Four studies added data for both EAOC and OCAE [10–13].

### Endometriosis-Associated Ovarian Cancer (EAOC)

Twenty-three studies of EAOC were selected, involving a total of 800 patients (SF2). In six studies, EAOC was defined as “ovarian cancer with endometriosis identified histologically in the same ovary, or endometriosis in one ovary and ovarian cancer in the contralateral ovary, or ovarian cancer with pelvic endometriosis” [11, 14–18]. In seven studies, the authors confirmed the coexistence of endometriosis and ovarian cancer in all cases [12, 19–24]. In the other studies, the presence of endometriosis with ovarian cancer was revealed by histopathological reports [10, 13, 25–32]. The mean age, if available, ranged from 41.1 to 58 years. Menopausal status was reported in six studies: in all but one (17), more than 50% of the women were premenopausal.

As shown in Table 1, of the 800 patients with endometriosis-associated ovarian cancer, 37.8% (95% CI 34.5–41.2) had clear cell carcinoma, 36.8% (95% CI 33.5–40.2) had endometrioid carcinoma, 4% (95% CI 2.8–5.6) mixed, 12.9% (95% CI 10.7–15.4) serous and 3.6% (95% CI 2.5–5.2) had mucinous carcinoma. Other histologies were less common. The FIGO stage of ovarian cancer was reported in 17 studies: from 45.5 to 100% of cases were stages I and II. The presence of atypical endometriosis was reported in five studies: in two studies, the frequency of atypical endometriosis was lower than 33% [14, 23], whereas in the other three studies, the presence of atypia ranged from 75 to 100% of cases [10, 16, 22] (see SF3a). Ogawa et al. observed the transition from typical to atypical endometriosis in 60% of cases and the transition from atypical endometriosis to carcinoma in 62% of cases. Only in one case, a direct transition from typical endometriosis to carcinoma was observed. Probably, the patients in whom the transition from endometriosis to carcinoma was observed could be considered OCAE, but as in the article the authors do not distinguish the frequencies of histological type in the two subpopulations, EAOC and OCAE, we consider all cases as EAOC, as reported in the article [22].

**Table 1:** Distribution of histological types of endometriosis-associated ovarian cancer (EAOC)

First author, year (ref)	Country	*n* EAOC	Clear cell ca	Endometrioid ca	Mixed	Serous ca	Mucinous ca	Other histologies	Stage FIGO (%)			
									I	II	III	IV
Acien, 2015 [14]	Spain	12	3	2	7							
Aris, 2010 [15]	Canada	41	9	10		8	2	12				
Bas-Esteve, 2019 [25]	Spain	23	8	14		1			60.9	17.4	21.7	0
Boyraz, 2013 [26]	Turkey	45	17	15		6	4	3	51.1	8.9	40.0	0
Erzen, 2001 [20]	Slovenia	57	6	33		4	1	13	67.2	19.0	12.1	1.7
Fukunaga, 1997 [10]	Japan	18	12	3		3			88.9	0	11.1	0
Jimbo,1997 [21]	Japan	25	13	3		8	1		68.0		32.0	
Ju, 2019 [15]	Korea	40	15	15		3	6	1	73.0	15.0	13.0	0
Kawahara, 2021 [27]	Japan	47	31	14		2						
Kondi-Pafiti, 2012 [28]	Greece	17	10	6		1			37.5	43.8	12.5	6.3
Lu, 2017 [11]	China	39	17	6		13^a^	3		43.6	20.5	33.3	2.6
Modesitt, 2002 [12]	USA	33	7	8	4	10		4	27.3	18.2	33.3	3
Ogawa, 2000 [22]	Japan	37	30	3		4			56.8	13.5	16.2	13.5
Oral, 2018 [16]	Turkey	32	4	15	3	7	2	1	69.0			
Qiu, 2013 [17]	China	17	8	6		3						
Sarmadi, 2018 [23]	Iran	22	6	15				1				
Stasienko, 2015 [24]	Poland	58	13	33		11	1		56.8		43.1	
Stern, 2001 [13]	USA	20	7	7		5	1					
Surprasert, 2006 [29]	Thailand	36	17	11	4	1	1	2	64.9	10.8	13.6	2.7
Udomsinkul, 2020 [30]	Thailand	79	48	26	5				59.7	20.8	13.0	6.5
Vercellini, 1993 [32]	Italy	63	8	30	8	8	6	3	65.1		28.6﻿	
Vercellini, 2000 [31]	Italy	22	5	13	1	2	1		100.0			
Wang, 2013 [18]	China	17	8	6		3			88.2	11.8		
	Total	800	302	294	32	103	29	40				
	%		37.8	36.8	4.0	12.9	3.6					
	95% CI		34.5–41.2	33.5–40.2	2.8–5.6	10.7–15.4	2.5–5.2					

*ref*, reference

*ca* carcinoma

^a^High grade

Overall survival at 5 years ranged from 76.9 to 80.3% and disease-free survival ranged from 55.4 to 77.6%, in the three studies that reported this data [11, 15, 25], while the Acien study showed, in invasive EAOC, a survival rate of just over 40% [14]. Moreover, a study did not demonstrate a difference in survival based on histology, whereas patients with stage III or IV cancer correlate with poor overall survival [12] (see SF3a).

### Ovarian Cancer Arising in Endometriosis (OCAE)

We also selected 12 studies in which ovarian cancer was defined as “arising in” endometriosis (Sampson and Scott’s criteria) (SF4). Three studies described ovarian cancer cases as “associated with” endometriosis, but since the authors defined cases as meeting Sampson and Scott’s criteria, we included them as OCAE [33–35]. Three studies also included cases of extra-ovarian cancer [12, 13, 36]. The mean age ranged from 41 to 54 years.

As shown in Table 2, of the 375 patients with OCAE, 43.5 (95% CI 38.5–48.5) had clear cell carcinoma, 35.7 (95% CI 31.1–40.7) endometrioid carcinoma, 2.7% (95% CI 1.5–4.8) mixed, 12.5% (95% CI 9.6–16.3) had serous and 2.4% (1.3–4.5) mucinous carcinoma. Seven studies reported FIGO stage: from 36.0 to 89.0% were stage I.

**Table 2:** Distribution of histological types of ovarian cancer arising in endometriosis (OCAE)

First author, year (ref)	Country	*n* OCAE	Clear cell ca	Endometrioid ca	Mixed ec ccc	Serous ca	Mucinous ca	Other histologies	Stage FIGO (%)			
									I	II	III	IV
Akbarzadeh-Jahromi, 2020 [35]	Iran	24	6	8		10						
Fishman, 1996 [37]	USA	8		6	2				62.5	25.0	12.5	0.0
Fukunaga, 1997 [10]	Japan	11	6	4		1			76.7		27.3	0.0
Heaps, 1990 [36]	USA	10	1	8				1				
Kawaguchi, 2008 [33]	Japan	18	11	6			1		89.0	11.0	0.0	0.0
Kumar, 2011 [34]	USA	42	9	6		23	4		45.0	2.0	38.0	12.0
Lai, 2013 [38]	Taiwan	79	40	33		4		2				
Lu, 2017 [11]	China	110	78	25	1	2	1	3	85.5	4.5	9.1	0.9
Modesitt, 2002^a^ [12]	USA	25	6	7	5	5		2	36.0	12.0	32.0	0.0
Prefumo, 2002 [39]	Italia	14	0	14					78.6	21.4	0.0	0.0
Stern. 2001^a^ [13]	USA	3	2	1								
Zanetta, 2000^b^ [40]	USA	31	4	16	2	2	1	4				
	TOTAL	375	163	134	10	47	9	12				
	Percentage		43.5	35.7	2.7	12.5	2.4					
	95% CI		38.5–48.5	31.1–40.7	1.5–4.8	9.6–16.3	1.3–4.5					

*ref*, reference

*ca*, carcinoma

^a^Extra-ovarian cancer was excluded

^b^The cancer arose in the ovary in 15 patients, in the serosa of the bowel in 5, in the cul-de-sac in 4, on the remaining serosa of the pelvis in 4, in the vagina in 2 and in the tube in 1. The study did not specify the site of the cancer in each histology

Only four studies reported the presence of atypical endometriosis: Fukunaga and Prefumo both documented the presence of severe atypia in 91 and 100% of ovarian cancer cases arising in endometriosis respectively [10, 39]. In Fukunaga’s study, this type of lesion showed histological contiguity with neoplasm [10]. The overall survival at 5 years varied from 62 to 91.2%, but only two studies reported this information (SF3b) [11, 34].

### Meta-analysis

There were no significant differences (*p*-value = 0.3376) in the distribution of the different histotypes of epithelial ovarian cancer in the two populations analysed: EAOC and OCAE.

**Fig. 2:**
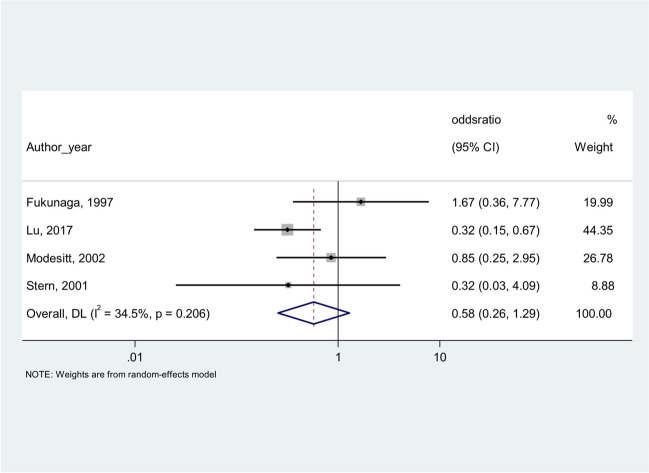
Clear cell carcinoma in EAOC versus clear cell carcinoma in OCAE

**Fig. 3:**
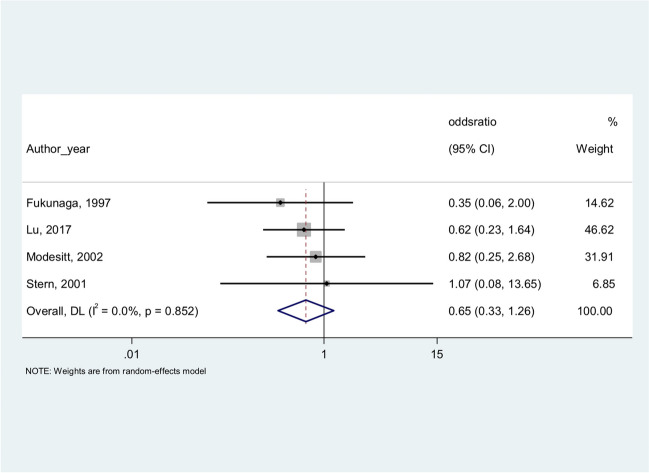
Endometrioid carcinoma in EAOC versus endometrioid carcinoma in OCAE

Four of the selected studies reported both EAOC and OCAE and their histological type distribution. When we performed a meta-analysis to compare the frequency of CCC and EC in EAOC and OCAE, both the histotypes of CCC and EC were less frequent in EAOC compared to OCAE, as shown in Fig. 2 for CCC (OR = 0.58 (0.26–1.29)) and in Fig. 3 for EC (OR = 0.65 (0.33–1.26)).

The frequency of serous carcinoma was lower, 12.9% and 12.5% in EAOC and OCAE respectively.

## Discussion

The results of our review show no significant differences in the distribution of histotypes in two populations: EAOC and OCAE. Clear cell carcinoma and endometrioid carcinoma were the most common subtypes and they were present in similar proportions in both selected populations. When we performed the meta-analysis in four of the selected studies (because they reported the histological type distribution of both EAOC and OCAE), both CCC and EC histotypes were less common in EAOC than in OCAE, although the difference was not statistically significant. The frequency of serous carcinoma was low in both populations. The other histotypes were present in small proportion. The pathogenesis of the development of malignancy from endometriosis is controversial and partly unknown. Some authors have suggested that, in high percentage of cases, the pathological transformation begins with atypical endometriosis proliferation, which has a precancerous potential [3, 4, 10, 14, 41, 42]. The presence and the increase of atypical changes in endometriosis may allow the identification of endometrial lesions at risk of malignant transformation and thus provide clinicians with indications for closer and long-term surveillance.

Oestrogen stimulation, tumour suppressor gene inactivation and oxidative stress reaction have been reported and may contribute to ovarian cancer development by creating a microenvironment that favours the accumulation of sufficient genetic alterations leading to malignant transformation in endometriosis. The genes involved in endometriosis and epithelial ovarian cancer have been shown to play a role in the pathogenesis of malignant transformation [43]. PTEN loss may be an early event in cancer development from endometriosis [35], as well as genetic mutations affecting ARID1A and PIK3CA seem to play a role [3]. Although mutation or loss of function of the p53 tumour suppressor gene is rare in endometriosis, it may play an important role in malignant transformation and is the most common and important genetic event in the development of ovarian cancer. P53 alterations were found in atypical endometriosis, whereas they were low or absent in non-atypical endometriosis. P53 mutation or accumulation was more common in endometrioid and serous carcinoma [38, 44]. In addition, the p53 tumour suppressor pathway may be important in the development of clear cell carcinoma [45]. Furthermore, chronic endometriosis-induced inflammation and the accumulation of genetic alterations contribute to and promote the progression to a malignant endometriosis-associated carcinoma phenotype. All these mechanisms could be of different magnitudes in the development of the different histotypes of ovarian carcinoma but be common to both associated and arising cases, leading to the predominance of clear cell carcinoma and endometrioid carcinoma, both in EAOC and OCAE. However, this topic is open to debate: recently, some authors have suggested that the origin and the relationship with endometriosis may be different in clear cell and endometrioid carcinoma [42].

The heterogeneity of epithelial ovarian cancer in relation to endometriosis makes the hypothesis of a new therapeutic approach challenging, but further studies are needed to understand the malignant transformation or association of ovarian cancer and endometriosis and thus identify new targets for the development of therapeutic interventions against EAOC or OCAE.

In the interpretation of our findings, some limitations should be considered: the demonstration of a histological transition between endometriosis and cancer requires extensive sectioning of the ovaries, which may not always be feasible. The histological examination of too few tumoural tissue sections could lead to an underestimation of OCAE and a selection bias between “associated” and “arising”. Thus, cases of ovarian cancer arising in endometriosis may have been considered “associated” because the transition from endometriosis to cancer, even if present, was not identified (Scott’s criterion).

In conclusion, this analysis shows that the histological profiles of EAOC and OCAE are similar, suggesting a similar aetiopathological mechanism, which requires further research to investigate whether EAOC and OCAE may be in the same way, but at different points, of transformation to malignancy or have different pathways of progression to malignancy.

### Supplementary Information

Below is the link to the electronic supplementary material.

Supplementary file1 (DOCX 45 KB)

Supplementary file2 (DOCX 21 KB)
Supplementary file3 (SF3a-SF3b)(DOCX 22.2 KB)

﻿Supplementary file4  (DOCX 17.3 KB)

## Data Availability

All data of the analysis are in the article.
