# Correction: Histologic Subtypes in Endometriosis-Associated Ovarian Cancer and Ovarian Cancer Arising in Endometriosis: A Systematic Review and Meta-Analysis

**DOI:** 10.1007/s43032-024-01556-1

**Published:** 2024-04-22

**Authors:** Francesca Chiaffarino, Sonia Cipriani, Elena Ricci, Giovanna Esposito, Fabio Parazzini, Paolo Vercellini

**Affiliations:** 1 Gynaecology Unit, Foundation IRCCS Ca’ Granda Ospedale Maggiore Policlinico, Via Commenda 12, Milan, 20122 Italy; 2 Department of Clinical Sciences and Community Health, University of Milan, Milan, Italy

**Reproductive Sciences**

10.1007/s43032-024-01489-9

The original online version of this article was revised to add the following funding notice: This study was partially funded by Italian Ministry of Health, Current Research IRCCS Ca’ Granda Ospedale Maggiore Policlinico, Milano.

The original article has been corrected.

