# Supplementary material for: Histologic Subtypes in Endometriosis-Associated Ovarian Cancer and Ovarian Cancer Arising in Endometriosis: A Systematic Review and Meta-Analysis
Source: Reprod Sci. 2024 Mar 4;31(6):1642–50. doi: 10.1007/s43032-024-01489-9 (PMC11111532; doi:10.1007/s43032-024-01489-9)
Supplement: Supplementary file 1 — Supplementary file1 (DOCX 45 KB) [file 43032_2024_1489_MOESM1_ESM.docx]

Supplementary File 1. SF1.

2020 flow diagram for study selection.

**Identification of studies via databases**

Records removed *before screening*:

Duplicate records removed (n = 1361)

Records identified from:

Pubmed (n=1412)

Embase (n=1804)

**Identification**

Records screened

(n = 1855)

Records excluded

(n =1721)

Reports sought for retrieval

(n =134)

Reports not retrieved

(n =2)

**Screening**

Reports excluded due to different reasons: review, case-reports, selected population, selected diagnosis, self-reported endometriosis, no useful data (n =101)

Reports assessed for eligibility

(n =132)

Studies included

(n=31).

**Included**

*From:*  Page MJ, McKenzie JE, Bossuyt PM, Boutron I, Hoffmann TC, Mulrow CD, et al. The PRISMA 2020 statement: an updated guideline for reporting systematic reviews. BMJ 2021;372:n71. doi: 10.1136/bmj.n71

For more information, visit: <http://www.prisma-statement.org/>
