# Supplementary material for: Histologic Subtypes in Endometriosis-Associated Ovarian Cancer and Ovarian Cancer Arising in Endometriosis: A Systematic Review and Meta-Analysis
Source: Reprod Sci. 2024 Mar 4;31(6):1642–50. doi: 10.1007/s43032-024-01489-9 (PMC11111532; doi:10.1007/s43032-024-01489-9)
Supplement: Supplementary file 2 — Supplementary file2 (DOCX 21 KB) Supplementary file3 (SF3a-SF3b)(DOCX 22.2 KB) [file 43032_2024_1489_MOESM2_ESM.docx]

| Supplementary File 2. SF2  Characteristics of selected studies on endometriosis associated ovarian cancer (EAOC) | | | | | |  |  |  |
| --- | --- | --- | --- | --- | --- | --- | --- | --- |
| **First Author, Years** | **Country** | **Study Design** | **Period** | **Age** | **Endometriosis Diagnosis** | **Definition Of EAOC** | **N EAOC** | **Notes** |
| Acien, 2015 | Spain | clinical series or retrospective study | 1993-2013 | 48.8 ±11.6 | histology | presence of ovarian cancer and E.^a^ in the same or controlateral ovary or extraovarian pelvic E:. | 12 | 4 cases were atypical E. +8 borderline |
| Aris, 2010 | Canada | retrospective cross-sectional based on registry | 1997-2006 | 48.3 ±10.8 | pathological report | pathological reports confirm the presence of ovarian cancer and E. | 41 |  |
| Bas-Esteve, 2019 | Spain | retrospective study | 1995-2015 | 50.9±11.2 | histology | from histopathological reports | 23 | + 13 borderline |
| Boyraz, 2013 | Turkey | retrospective study | 2000-2010 | 55.0 | histology | from histopathological reports | 45 | 89% had high grade carcinoma |
| Erzen, 2001 | Slovenia | nested case-control study | 1990-1999 | 54.5 ±11.5 | histology | ovarian E. in 79%, uterus 17%,extrauterine 3%. In 38% of women E. had been diagnosed before carcinoma (stage3-4) but coexistence was histologically confirmed in all cases. | 57 | Borderline cases were excluded from the study. |
| Fukunaga, 1997 | Japan | consecutive patients | 1987-1995 | 51.3 | histology | cases fulfilled the Sampson's criteria | 18 | In all of cases atypical E. was present (100%). Borderline cases were excluded. |
| Jimbo,1997 | Japan | clinical series or retrospective study | 1980-1995 |  | histology | coexistence of ovarian E. confirmed by presence of glandular epithelium accompanied by endometrioid stroma in the ovaries. | 25 | 13 % were premenopausal |
| Ju, 2019 | Korea | retrospective study | 2004-2016 | 47.2 ± 13 | histology | ovarian cancer with E. identified histologically in the same ovary, or E. in one ovary and ovarian cancer in the contralateral ovary, or ovarian cancer with extra-ovarian pelvic E. | 40 | 35% grade 1. 65% were premenopausal |
| Kawahara, 2021 | Japan | retrospective study | 2012-2019 |  |  | magnetic resonance confirmed E. associated ovarian cancer | 47 |  |
| Kondi-Pafiti, 2012 | Greece | retrospective study | 2000-2009 | 58 | E. was documented in 47% of the tumors | In 53% fibrotic tissue with pseudoxanthoma cell and few residual stromal cells were considered strong evidence of E. | 17 |  |
| Lu, 2017 | China | retrospective study | 1995-2014 | 49 ± 8,8 | histology | presence of ovarian carcinoma and E. in the same ovary or presence of ovarian carcinoma in one ovary and E. in the contralateral ovary or presence of ovarian carcinoma and extra-ovarian E, without continuity between them | 39 |  |
| Modesitt, 2002 | USA | retrospective study | 1970-1999 |  | histology | ovarian cancer with adjacent E. | 33 | 67% were premenopausal. 24% contraceptive use. 15% HRT use |
| Ogawa, 2000 | Japan | retrospective study | 1980-1995 | 48.9 | histology | presence of typical or atypical E. and carcinoma were histologically evaluated | 37 | 78% showed atypical endometriosis |
| Oral, 2018 | Turkey | retrospective study | 1995-2011 | 49.2± 10.4 | histology | ovarian cancer with E. identified histologically in the same ovary, or E. in one ovary and ovarian cancer in the contralateral ovary, or ovarian cancer with pelvic E. | 32 | 75% showed atypical E.. 52% were premenopausal |
| Qiu, 2013 | China | retrospective study | 2011-2012 |  | histology | ovarian cancer with E: identified histologically in the same ovary, or E: in one ovary and ovarian cancer in the contralateral ovary, or ovarian cancer with pelvic E: | 17 | E: present in the ovaries or peritoneum |
| Sarmadi, 2018 | Iran | retrospective study | 2007-2014 | 41.1 | histology | inclusion criterion was coexistence of E: and ovarian cancer in one post-operative tissue specimen | 22 | Atypical E: in 4 cases |
| Stasienko, 2015 | Poland | retrospective study | 2004-2010 |  | histology | inclusion criterion was coexistence of E: and ovarian cancer in one post-operative tissue specimen | 58 |  |
| Stern, 2001 | USA | consecutive patients | 1980-1999 |  | histology. SNOMED code | cancer was in ovary but not continuous with the E: | 23 |  |
| Surprasert, 2006 | Thailand | retrospective study | 1996-2005 | 44 | histology | Medical record of ovarian cancer with histological evidence of E:. 27% had pelvic E:, 6% E: in controlateral ovary | 36 |  |
| Udomsinkul, 2020 | Thailand | retrospective study | 1999-2014 | 49 |  | endometriotic cyst associated ovarian cancer | 79 | 53% were premenopausal |
| Vercellini, 1993 | Italy | retrospective study | 1980-1991 |  | histology | from pathological reports | 63 |  |
| Vercellini, 2000 | Italy | clinical record of consecutive women | 1992-1999 |  | histology | from pathological reports | 22 | 3 patients with endometrioid cancer had concomitant endometrial cancer |
| Wang, 2013 | China | retrospective study | 2011-2012 | 46.1 ± 10.1 |  | ovarian cancer with E: identified histologically in the same ovary, or E: in one ovary and ovarian cancer in the contralateral ovary, or ovarian cancer with pelvic E: | 17 | 77% were premenopausal |

E.: endometriosis
