# Supplementary material for: Histologic Subtypes in Endometriosis-Associated Ovarian Cancer and Ovarian Cancer Arising in Endometriosis: A Systematic Review and Meta-Analysis
Source: Reprod Sci. 2024 Mar 4;31(6):1642–50. doi: 10.1007/s43032-024-01489-9 (PMC11111532; doi:10.1007/s43032-024-01489-9)
Supplement: Supplementary file 3 — ﻿Supplementary file4 (DOCX 17.3 KB) [file 43032_2024_1489_MOESM3_ESM.docx]

**Supplementary File 3a. SF 3a**

Other characteristics of selected studies on endometriosis associated ovarian cancer (EAOC)

|  | **survival** | **atypical^a^** | **grade** | **treatment** |
| --- | --- | --- | --- | --- |
| Acien,2015 | overall survival in 5 years was a bit more 40% and in 10 years was 20% | 4/12=33.3% |  |  |
| Aris,2010 | survival analysis included the borderline tumors (40% of cases) that we excluded |  |  |  |
| Bas_esteve, 2019 | survival at 2 years=84.6%, at 5 years=76.9%; at 10 years=65.9%. Survival was greater than 90% at 10 years in stage I and II with associated endometriosis. | atypical or p53+: 8/36(including borderline)=22% | n.a. |  |
| Boyraz,2013 | overall survival after a median follow-up of 42 months was 64.4% (29/45) |  | Low grade: 5 (11.1%) High grade:40 (88.9%) |  |
| Erzen, 2001 | overall survival at the end of follow-up (46.6 ±30.7 months) was 81 %. Stage specific (FIGO) survival: stage 1: 89.7%, stage II: 72.2%, stage III: 42.9% |  | grade I:22 (37.9%) grade II: 24 (41.4%) grade III:12 (20.7%) |  |
| Fukunaga, 1997 | n.a. | 18/18=100% |  |  |
| Jimbo, 1997 | n.a. |  |  |  |
| Ju,2019 | After a median follow-up of 32 month (range 6-156 months), the 5 years disease-free survival rate was 77.6% and the overall survival rates was 80.3% |  | grade 1: 14 (35%) grade 2: 5 (13%) grade 3: 21 (53%) | All women underwent primary surgery. Of these, 35 (88%) received adjuvant chemotherapy. During the follow-up period, nine women had a recurrence, and eight of them died owing to disease progression. |
| Kawahara,2021 | niente |  |  |  |
| Kondi - Pafiti 2012 | No disease recurrence or death of a patient was noted during the time of the follow-up which ranged from 12–24 months. |  | All of the patients had both ovarian and endometrial stage I tumors, mostly low grade. |  |
| Lu, 2017 | After a median follow-up of 47 months (range, 2-224 months) the overall survival in 5 years was 79.1% . The 5-year disease-free survival was 70.4 % | n.a. |  | All patients underwent surgery, then received chemotherapy. |
| Modesitt, 2002 | Among the group as a whole (endometriosis associated intraperitoneal cancer,n=115), 61 patients (55%) had a recurrence of cancer, the median disease-free interval for the entire group was 21 months, and median survival was 35 months. Histology and chemotherapy type were no significantly associated with survival. This study did not demonstrate a difference in survival based on histology. Survival data demonstrated that patients with stage III or IV tumors, grade 3 tumors, serous or clear cell type histology, and a history of two or more pregnancies and who received platinum postoperative chemotherapy correlated with a poor overall survival. | n.a. | grade 1=15.2%; grade 2=24.2%; grade 3=48.5% | Postoperative therapy was given in 61% of cases of grade 1 tumors, compared with 87% of grade 2 tumors and 97% of grade 3 tumors. Similarly, 73% of patients with stage I tumors and 98% of stages II, III, and IV patients received postoperative treatment. |
| Ogawa, 2000 | n.a. | 29/37=78.4% |  | All the patients were initially treated by surgery. Following surgery, combination chemotherapy was administered. |
| Oral, 2018 | n.a. | 75% of cases | n.a. | n.a. |
| Qiu, 2013 | n.a. | n.a. | 15 (%) type I; 2(%) type II | n.a. |
| Sarmadi, 2018 | n.a. | 4/22=18.2% |  |  |
| Stasienko,2015 | n.a. | n.a. | grade 1=41.3%; grade 2=12.1%; grade 3=46.6% | n.a. |
| Stern,2001 | n.a. | n.a. | n.a. | n.a. |
| Surprasert, 2006 | The estimated 5-year disease-free survival was 55.4%, with a mean follow up time of 73 months (3-117 months) | n.a. | n.a. | n.a. |
| Udomsinkul,2020 | n.a. | n.a. | n.a. | all of patients underwent surgery and 94% had adjuvant chemotherapy |
| Vercellini, 1993 | n.a. | n.a. | n.a. |  |
| Vercellini, 2000 | n.a. | n.a. | n.a. |  |
| Wang, 2013 | n.a. | n.a. | n.a. |  |
|  |  |  |  |  |
|  | EAOC: endometriosis associated ovarian cancer |  |  |  |
|  |  |  |  |  |
|  | a: n atypical endometriosis/total EAOC=% |  |  |  |

**Supplementary File 3b. SF 3b**

Other characteristics of selected studies on ovarian cancer arising endometriosis (OCAE)

|  | **survival** | **atypical^a^** | **grade** | **treatment** |
| --- | --- | --- | --- | --- |
| Akbarzadeh-Jahromi, 2020 | n.a. | n.a. | n.a. | n.a. |
| Fishman, 1996 | At 12 months 100% alive | 7/8=87.5% | grade 1=25%; grade 2=75%; | 75% chemotherapy |
| Fukunaga, 1997 | n.a. | 10/11=91% | n.a. | n.a. |
| Heaps, 1990 |  |  |  |  |
| Kawaguchi, 2008 |  | 2/18=11.1% | n.a. | 94% chemotherapy |
| Kumar, 2011 | 62% survival at 5 years. Median survival was 16.5 years |  |  |  |
| Lai, 2013 | n.a. |  | n.a. | n.a. |
| Lu, 2017 | After a median follow-up of 47 months (range, 2-224 months), the overall survival in 5 years was 91.2% . the 5-year DFS was 87.6% |  | n.a. | n.a. |
| Modesitt, 2002 | Among the group as a whole (endometriosis associated intraperitoneal cancer,n=115), 61 patients (55%) had a recurrence of cancer, the median disease-free interval for the entire group was 21 months, and median survival was 35 months. Histology and chemotherapy type were no significantly associated with survival. This study did not demonstrate a difference in survival based on histology. | n.a. | grade 1=36%; grade 2=16%; grade 3=44% |  |
| Prefumo, 2002 | n.a. | 14/14=100% | grade 1=57.1%; grade 2=42.9%; |  |
| Stern. 2001 | n.a. |  | n.a. | n.a. |
| Zanetta, 2000 | n.a. | n.a. | grade1=6 endometrioid |  |
|  | OCAE: ovarian cancer arising endometriosis |  |  |  |
|  |  |  |  |  |
|  | a: n atypical endometriosis/total OCAE=% |  |  |  |
